# Morphometric approaches to *Cannabis* evolution and differentiation from archaeological sites: interpreting the archaeobotanical evidence from bronze age Haimenkou, Yunnan

**DOI:** 10.1007/s00334-023-00966-6

**Published:** 2023-11-30

**Authors:** Rita Dal Martello, Rui Min, Chris J. Stevens, Ling Qin, Dorian Q. Fuller

**Affiliations:** 1 Department of Asian and North African studies, Ca’ Foscari University, Dorsoduro 3462, 30123 Venice, Italy; 2 Domestication and Anthropogenic Evolution Research Group, Max Planck Institute of Geoanthropology, Kahlaische Straße 10, 07745 Jena, Germany; 3 Yunnan Province Institute for Cultural Relics and Archaeology, Kunming, 650118 China; 4 Institute of Archaeology, University College London, 31-33 Gordon Square, London, WC1H 0PY UK; 5 Department of Archaeology, Cambridge University, Downing Street, Cambridge, CB2 3DZ UK; 6 School of Archaeology and Museology, Peking University, Yiheyuan Rd. 5, Haidian District, Beijing, 10087 China; 7 School of Cultural Heritage, Northwest University, Xi’an, 710127 Shaanxi China

**Keywords:** Archaeobotany, Domestication, Hemp, Marijuana, Cannabis, China

## Abstract

Cannabis grains are frequently reported from archaeological sites in Asia, and hypothesized centers of origins are China and Central Asia. Chinese early cannabis remains are often interpreted as evidence of hemp fabric production, in line with early textual evidence describing ritualistic hemp cloth use and hemp cultivation as a grain crop. Modern measurements on cannabis varieties show distinct sizes between fibre or oil/fibre and psychoactive varieties, the former having larger seeds on average than the latter. This paper reviews the current macro-botanical evidence for cannabis across East, Central and South Asia and builds a comparative framework based on modern cannabis seed measurements to help identify cannabis use in the past, through the metric analysis of archaeologically preserved seeds. Over 800 grains of cannabis were retrieved from the 2008 excavation of Haimenkou, Yunnan, Southwest China, dating to between 1650 and 400 bc. These are compared with other known archaeological cannabis and interpreted through the metric framework. This offers a basis for exploration of the seed morphometrics potential to infer cannabis cultivation and diversification in uses. At Haimenkou, cannabis seeds size mostly plot in the range of overlapping psychoactive/fibre types; we therefore suggest that the cannabis assemblage from Haimenkou is indicative of a crop beginning to undergo evolution from its early domesticated form towards a diversified crop specialized for alternative uses, including larger oilseed/fibre adapted varieties.

**Supplementary Information:**

The online version contains supplementary material available at 10.1007/s00334-023-00966-6.

## Introduction

Cannabis is the source of the well-known drugs marijuana and hashish, hemp fibres used for cloth and rope, and edible oilseeds. Its domestication, early history of cultivation and diversification are presently poorly understood. Although not a staple grain today, cannabis was considered one of ancient China’s “five grains”, with millet, rice, barley, and soybean (Huang 2000). However, its presumed secondary role in the overall subsistence makes its seeds less likely to be processed in bulk and turn up in archaeobotanical assemblages when compared to cereals or pulses. *Cannabis sativa* (*sensu lato*) was probably selected early on for multiple uses as a fibre and an oilseed, as well as for medicinal/ritual drug uses. It might therefore be considered as an East Asian example comparable to the multi-use crop flax (*Linum usitatissimum*) that was cultivated alongside wheat and barley in early western Asia. The wild progenitor(s) of cannabis cultivars may be unknowable; today, naturalized cannabis plants that escape cultivation show wild-type characters in as little as 50 years (Small 1975) and it is posited that the native distribution area of this plant was broadly distributed in eastern Eurasia (from eastern Europe to Japan). Legal controls over the cultivation and transport of some cannabis varieties have meant that genomic datasets remain somewhat limited (see Hillig 2005). Nevertheless, there are recent taxonomic syntheses (Clarke and Merlin 2013; McPartland and Small 2020) and a growing archaeobotanical record (Jiang et al. 2016; Long et al. 2017; McPartland and Hegman 2018; Ren et al. 2019), which means we are in an improved position to deduce aspects of the domestication and differentiation process.

Although several types of cannabis remains have been found and reported archaeologically, including seeds (or achenes), seed impressions, pollen grains, fibres, textile fragments and impressions, and hemp paper, seeds have the advantage of providing the possibility of morphometric comparison, as well as attesting direct evidence for local cultivation; therefore, for this study, we focus on archaeologically reported cannabis seeds. In this paper, we present evidence for cannabis use from the Bronze Age site of Haimenkou, in northwest Yunnan Province, Southwest China (1600−400 bc), where high quantities of *Cannabis* sp. seeds were recovered in association with cereal remains, especially rice and millet (Xue et al. 2022). This provides a basis for consideration of the evolving uses of this plant in Yunnan and elsewhere in China, and an exploration of the potential for using seed morphometrics to infer cannabis cultivation and diversification for textile and/or oilseed use or for psychoactive/medicinal uses.

## Taxonomic background and theories on the origins of cannabis

Scholars have proposed cannabis originated either in Central Asia, based on Vavilovian principles of modern distribution of highly diverse cultivated populations and archaeological pollen analyses (i.e. Vavilov and Dorofeyev 1992; Russo 2007; Long et al. 2017; McPartland et al. 2019; Rull 2022), or Northern China, due to the relative high number and frequency of early archaeological finds (Chang 1986; Wu et al. 2003; Crawford 2006). Others have proposed that modern cannabis cultigens may also derive from multiple, independent domestications (Vavilov 1926; Clarke and Merlin 2013; McPartland and Hegman 2018; Rull 2022), however recent phylogenetic analyses have posited that cannabis originated in Southwest China (Zhang et al. 2018; McPartland et al. 2019; Ren et al. 2021). To some extent these alternative theories can be linked to taxonomic uncertainty and controversy.

Cannabis belongs to the Cannabaceae family, comprising ten extant genera and about 170 species in the Old World (APG 2003), the others being hops (*Humulus*), and according to recent phylogenetics, trees including hackberries (*Celtis* L.), *Aphananthe* Planch., *Chaetachme* Planch., *Gironniera* Gaudich., *Lozanella* Greenm., *Pteroceltis* Maxim., *Trema* Lour., and *Parasponia* Miq. (some scholars group *Parasponia* with *Trema*; see Simpson 2010; Kovalchuk et al. 2020). Many botanical sources follow the taxonomy of Small and Cronquist (1976), more recently (Small 2017) updated considering genetic studies, which recognize just one species, *Cannabis sativa* L. According to the monotypic view, *C. sativa* is further divided into ssp. *sativa* var. *spontanea*, representing all wild and weedy varieties, ssp. *sativa*, representing all hemp fibre and oilseed cultivars, and ssp. *indica*, representing all cultivars grown primarily for psychoactive properties (see also McPartland 2018, 2020; McPartland and Small 2020). Other scholars instead argue for three separate species including *C. sativa* L., *C. indica* Lam., and *C. ruderalis* (Hillig 2004; Sawler et al. 2015; Clarke and Merlin 2013; Henry et al. 2020). The monotypic vs. polytypic view of *Cannabis* taxonomy is highly debated among scholars, however, the ICN Code (Turland et al. 2018) recognizes just one species and therefore we follow this view and outline further details about relevant subspecies and varieties below.

1. *Cannabis sativa* L. (*sensu strictu*) includes both wild and domesticated forms.
2. *C. sativa* ssp. *sativa* var. *spontanea* Vav. (syn. *C. ruderalis* Janisch), the narrowleaf hemp-type taxon that includes extant European wild-like varieties. In general seeds of these plants are smaller in size compared to domesticated plants, and are expected to have natural seed shattering, where the achene is detached from the seed through the formation of an abscission zone, causing the seeds to have an elongated tapered base, and prominent abscission zone, which should make it archaeobotanically distinct from domesticated forms, but de Candolle (1885) reported the presence of this type of cannabis in the South Caspian region, among other areas. This taxon likely includes feral populations, and feral hybrids resulting from introgression between *sativa* and *indica* cultivars, and thus its distribution may be the product of recent anthropogenically facilitated gene flow (see Clarke and Merlin 2013, p. 317).
3. *C. sativa* ssp. *sativa*, narrowleaf hemp cultivars, mainly grown for fibre production, traditionally in Eastern Europe.
4. *C. sativa* var. *chinensis* (Delile) DeBeaux. Broadleaf hemp cultivars, an East Asian textile crop, and oilseed varieties. This is hypothesized to have been selected from ssp. *indica* for larger seeds and/or taller plants, and generally lower THC production.
5. *C. sativa* ssp. *indica* var. *asperrima* (Regel) McPart. & Small (syn. *C. indica* var. *kafiristanica* Vav.), the narrowleaf drug-type plants from Central Asia (McPartland and Small 2020); this variety was first described by Vavilov in the 1930s on the basis of weedy material in eastern Afghanistan. This wild taxon has significant THC production, and it is suggested to have expanded in the post-glacial period out of refugia in southwestern China, e.g. Hengduan Mountains and Yungui Plateau, i.e. Sichuan, Yunnan, Guizhou (Clarke and Merlin 2013, p. 325). This Post-Pleistocene expansion would have brought this species northward, to a point where it extended over much of northeast Asia in general. While an Indian refugium seems plausible no pollen evidence has yet been recorded to support this.
6. *C. sativa* ssp. *indica* var. *indica* (Lam.) Persoon (syn. *C. indica* ssp. *indica* (Lam.) Clarke and Merlin). Narrowleaf drug cultivars, including Indian “ganja”. Clarke and Merlin (2013) postulated that these may be the original eastern Asia domesticated form, which lost seed shattering and the basal seed caruncle. *C. sativa* ssp. *indica* var. *afghanica* (Vav.) McPart. & E.Small. Broadleaf drug cultivars, Central Asian hashish, used to produce drug resin. Selected for more vegetative growth and high THC (tetrahydrocannabinol, is the main psychoactive component).
7. *C. sativa* ssp. *indica* var. *himalayensis* (Cazzuola) McPart. & E.Small. Narrow-leaf drug type found in South Asia, especially the Himalayas (McPartland and Small 2020), typically used for hashish, seldom for seed oil.

The above taxonomy is linked to a set of evolutionary hypotheses in which wild populations were already structured into higher and lower THC varieties and inversely correlated levels of CBD (cannabidiol, which has a calming effect and medicinal uses in pain relief). For example, traditional varieties of hemp across China, are reported to range from 0.02 to 4.3% THC content by dry weight (Hong and Clarke 1996). Clarke and Merlin (2013) infer that wild forms of *asperrima*/*kafiristanica* may have encompassed considerable genetic diversity that included higher THC production. Nevertheless, early cultivars are likely to have been variable and not yet selected for specialized drug or fibre uses. The analysis of a worldwide genomic panel of cannabis by Ren et al. (2021) suggests that these two specialized uses diverged from ancestral general cultivars around the early second millennium bc (ca. 3800 bp). The same study posited domestication as early as the start of the Holocene (ca.12,000 bp; Ren et al. 2021), but the limited sampling of wild populations and wide error bars on such estimates calls for ground truthing such hypothesis through empirical archaeobotanical evidence. For example, the application of similar genetic methods to Asian rice estimated genetic divergence millennia to as much 10,000 years earlier than the first finds of domesticated archaeological remains (Choi et al. 2017). It also should be noted that there are no ancient genomic data to aid calibration of these timescales.

**Fig. 1:**
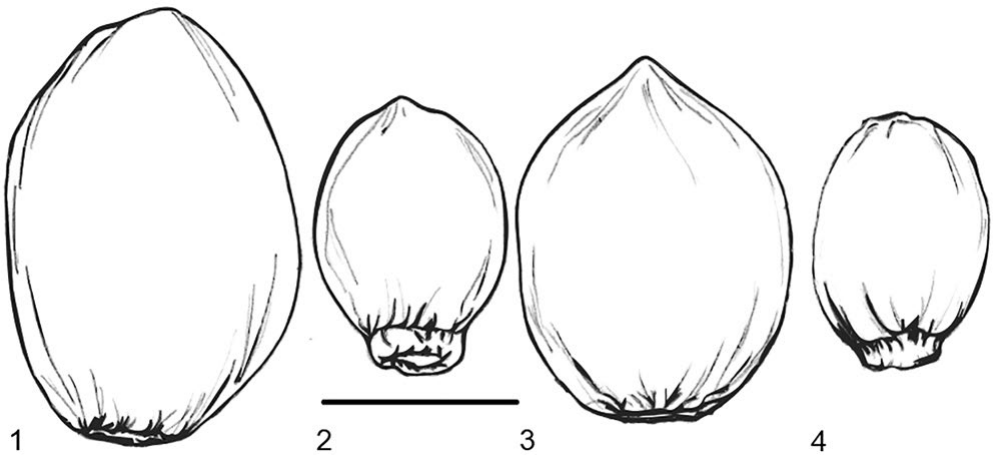
Drawings of modern cannabis seeds, adapted from Small and Cronquist 1976. 1 *C. sativa* ssp. *sativa* (hemp cultivar), 2 *C. sativa* ssp. *sativa* var. *spontanea* (narrow leaf hemp type ancestor), 3 *C. sativa* ssp. *indica* (drug cultivar), 4 *C. sativa* ssp. *indica* var. *asperrima/kafiristanica* (narrow leaf drug type ancestor), scale bar 2 mm. Note the distinctive protruding caruncle in the shattering wild varieties 2 and 4

Seed morphology, especially size, varies greatly across the cannabis complex, which offers scope for studying this aspect of evolution through archaeobotanical evidence. Presently more archaeobotanically oriented morphometric work is needed, but the caruncle presence and shape of the hilum/abscission zone do appear to vary between species and sub-species (Fig. 1; e.g. Small and Cronquist 1976; Clarke and Merlin 2013) and suggest that domestication and diversification may be amenable to archaeobotanical analysis. One axis of variation that is currently apparent is that larger seeds are typical of both hemp-fibre and edible oilseed varieties, while seeds of plants cultivated for drugs are generally smaller (Clarke and Merlin 2013, although some modern drug-type seeds can show larger achenes up to ≥ 3.6 mm long, see McPartland and Small 2020: Fig. 3). This pattern of seed size can be compared and contrasted to that of flax, in which early domesticates with larger seeds may have had more importance as oilseeds, while seed size decreased in the Bronze Age with selection for specialized fibre varieties (e.g. Herbig and Maier 2011). In the present contribution we assess existing seed morphometric evidence to assess the antiquity of distinctive fibre /oil seed and drug varieties in eastern Asian cannabis.

Cannabis is usually dioecious, having separate male and female plants, apart from plants with monoecious (intersexual) and hermaphroditic (bisexual) flowers. This enforces cross-pollination and maintains diversity within populations. Apart from males not producing seeds, male and female plants also differ by reaching ripeness at different stages, with male plants maturing five to six weeks earlier than females (Edwards and Whittington 1992). This makes the two sexes easily distinguishable and possibly allowed people in the past to select for particular variations, for example to weed out female plants in order to maintain male plants with preferred characteristics (for fibres) or selecting female plants for seeds or psychoactive use by potentially weeding out male plants. However, the dioecious nature of the plant makes it harder to fix selected traits in contrast to self-pollinating species, as are most early seed crops (Clarke and Merlin 2013). In addition to genetic diversity and heterozygosity encouraged by cross-pollination, cannabis is regarded to generally have high phenotypic plasticity (Russo 2007). As explored in Edwards and Whittington (1992) the intended use of cannabis will also impact preferences for the density of male plants maintained in fields. For drug purposes, female plants are preferred, seeds are less needed and so male plants may be fewer (thus less pollen production) given that for some production pollination is generally undesirable as it reduces the phytocannabinoids (Lipson Feder et al. 2021); this is even more so with plants reproduced by cloning. In contrast, for oilseed crops more male plants are needed to ensure all potential seeds are produced through pollination. Similarly, in fibre crops male plants are preferred for producing fibre, which may even be of better quality.

Cannabis grows well on most types of soils, but especially in high nitrogen content soils. The hypothesized original habitat for cannabis (inferred from the optimal growing conditions seen for ruderal cannabis) is a moist, but well drained, open sunny area with a high level of nitrogen in the soil. Growing near streams visited by mammalian herds could have provided the required high levels of nitrogen generated through their urine and dung (Clarke and Merlin 2013). This led to the hypothesis that cannabis was a “camp follower” and may have been first cultivated from volunteer plants on dumps near human habitation (Vavilov 1926; Anderson 1952, p. 167). In nature, cannabis is wind pollinated, however today, whereas cannabis plants bred for fibres and other uses are propagated from seeds or more recently through tissue culture methods (Ranalli 2004; Salentijn et al. 2015; Simiyu et al. 2022), plants bred for medicinal/psychoactive use are mostly cloned through vegetative propagation. Cannabis is particularly invasive of freshly disturbed soil areas (Small 2015), and as a result weedy forms have become widely distributed worldwide, being found on disturbed roadsides, by watercourses, and in cultivated fields. This provides ample scope for introgression that will inevitably complicate historical signals in genetic data.

In recent years, thanks to the increasing deployment of flotation for the recovery of archaeobotanical material during archaeological excavation in Asia, an increasing number of cannabis remains, especially seeds, have been recovered in many early sites across China. Textiles may also be preserved, but accurate identification of bast fibres to species is difficult (e.g. see Catling and Grayson 1982) and rarely reported with convincing details, while claims of identifying hemp textiles from superficial impressions, e.g. on ceramics (McPartland and Hegman 2018; cf. Merlin 2003) are problematic. Pollen has also been widely used to identify the past presence of cannabis. However, wild versus cultivated cannabis cannot be distinguished through pollen. In addition, *Cannabis* sp. and *Humulus* sp. (hops) produce morphologically similar pollen grains, which may lead to mistaken identification from the archaeological record (Lewis et al. 1983). The genus *Humulus* includes widespread, and often weedy, vines native to both Europe (*H. lupulus* L.) and Asia (*H. scandens* Lour., syn. *H. japonicus* Siebold & Zucc., with more localized *H. yunnanensis* Hu). Cultivated hops, used widely in beer brewing, are cultivars selected from European *H. lupulus* since the 9th century (Behre 1999). Aside from positively identified textiles of hemp, cannabis seeds from archaeological sites are seen as among the most ubiquitous and reliable indicators of past human use, as opposed to pollen, which indicates local presence of cannabis plants but does not provide information on whether plants were necessarily exploited or cultivated (Long et al. 2017; McPartland et al. 2019; Rull 2022).

### Written records on the antiquity and use of *Cannabis* in China

In early Chinese written texts, cannabis is referred to as *má* 麻 and most often translated as hemp, implying its use as a fibre plant. The earliest written accounts of cannabis cultivation and use date to the 1st millennium bc (see Table 1, ESM 1 Table S1 for full quotes and translations). It must be noted however that *má* also became a generic term for a bast fibre or other oil plants, with other kinds of *má* specified, such as *zhùmá* 苎麻 for ramie (*Boehmeria nivea* (L.) Guadich), *xúnmá* 荨麻 for nettles (*Urtica* spp.), or *zhīmá* 芝麻 for sesame (*Sesamum indicum* L.). Nevertheless, early occurrences of *má* as cannabis include poems in the *Shī Jīng* (Book of Odes), where there is a description of how and when to plant cannabis, while descriptions of hemp cloths are recorded in the *Shàng Shū* (Book of Historical Documents), and the *Lǚshì Chūnqiū* (Master Lü’s Spring and Autumn Annals). In the *Lĭ Jì* (Book of Rites), hemp headbands are prescribed to be worn to honor the dead during mourning activities. In the *Zhōu Lĭ* (the Rites of Zhou), cannabis is grouped with other cereals, including rice, millets, wheat/barley and soybean, attesting to its dual use as fibre and food grain. That cannabis is often described in this and the other works as being cultivated with other cereals, such as millet and wheat, has been interpreted as clear indication of its culinary use. The inferred use of cannabis as food grain is also supported by definitions given in several *Běncăo* (Chinese traditional Materia Medica), written from the early Eastern Han Dynasty onward in the early first millennium ad (ca. ad 1–200, Brand and Zhao 2017). Within the *Běncăo*, cannabis is classified as a *gŭ*, “grain” food crop, together with rice, millets, wheat, and others (Li 2005). The first written evidence relating to a medicinal use of cannabis is found in the *Shénnóng Běncăo Jīng*, Divine Farmer’s Classic of Materia Medica, traditionally dated to the Western Han Dynasty (first to second centuries ad; Li 1974; Touw 1981). According to this wealth of written evidence, we know that cannabis was known and widely employed in early Chinese societies from at least the first millennium bc, and that the versatile nature of the plant was also understood.

**Table 1:** Written accounts of cannabis use from early Chinese texts (see ESM 1 Table S1 for full quotes and translations)

Historical text	Date (bc)	Inferred use	References
*Shī Jīng* Book of Odes	c. 1000 − 700	Grain, description of planting times and cultivation systems	Legge (1876), (1879), Li( 1974), Huang (2000)
*Zhōu Lĭ* The Rites of Zhou	c. 1000 − 700 (written at end of 1st mill. bc)	Fibre and grain; cannabis grouped with millets, barley, rice, soybean and other plants referred to as the nine *gu* (meaning grain)	Biot (1851), Li (1974 p. 443)
*Shàng Shū* Book of Historical Documents	c. 772 − 476	Fibre	Legge (1879)
*Lĭ Jì* The Classic of Rites	c. 475 − 221	Fibre	Legge (1885), Li (1974)
*Lǚshì Chūnqiū* Master Lü’s Spring and Autumn Annals	c. 247 − 239	Fibre	Li (1974), Knobock and Riegel (2000)
*Shì Jì* Records of the Grand Historian	c. 109 − 91	Fibre; grain	Qian (1993)
*Shénnóng Běncăo Jīng* Divine Farmer’s Classic of Materia Medica	1st-2nd centuries ad	Psychoactive/medicinal use	Li (1974 p. 446), Yang (1998), Liu (1999), Lu and Clarke (1995)
Ĕr Yă zhù Approach to correct expression	3rd-4th centuries ad	Dioecious description of the plant (male and female plants)	Clarke and Merlin (2013 p. 141), Gao (1996)

Finally, the first clear written reference to male and female cannabis plants is found in the *Ĕr Yă zhù*, a commentary by Guō Pú (ad 276–324; see Gao 1996; Clarke and Merlin 2013), based and expanded upon the earlier *Ĕr Yă* dictionary (itself dated to the Han Dynasty, ca. 206 bc-ad 220; see ESM 1 Table S1).). This commentary refers to cannabis by indicating whether the plant produces seeds, calling it *mámù* 麻母, or if it does not produce seeds calling it *xĭ*枲. Later scholars have interpreted *xĭ* as male cannabis (for hemp production) and *mámu* as female cannabis (for other uses). This differentiation in male and female plants is seen as indication of an understanding of the dioecious nature of the plant possibly tied with different specific uses (Li 1974; Huang 2000; Clarke and Merlin 2013, p. 203).

## Materials and methods

### Archaeological and archaeobotanical research at Haimenkou

Haimenkou lies in the Jinsha (Yangtze) river basin at 2,190 m above sea level, in Jianchuan County, northwest Yunnan (26.466914 N, 99.919778 E; Min 2013). This is a mountainous area with distinct dry and wet seasons (between May and October, and November and April, respectively), and an average annual precipitation of 1,000–1,200 ml. After its initial discovery in 1957, Haimenkou underwent several excavation campaigns (YPM 1958; Xiao 1995; YPICRA et al. 2009). The site represents the largest prehistoric site discovered so far in Yunnan, extending over ~ 5 ha (Yao 2010). Large, rectilinear pile dwellings with wooden postholes preserved by waterlogging characterize the site, and the material culture retrieved includes small bronze objects, lithics and bone tools, and ceramic remains (Li and Min 2014). A textile fragment was recovered during the 2008 excavation (Xue et al. 2022: suppl. Material S4F); however no further study or analysis has been carried out so far in order to identify the fibres. Over the course of the 2008 excavation season, archaeobotanical samples for flotation were collected. Laboratory analyses of these samples revealed a flourishing productive economy based on the cultivation of rice and millet for the initial phase of occupation (ca. 1600−1400 bc), followed by the introduction of wheat from ca. 1400 bc, and its increasing importance in the last period of occupation (ca. 800−400 bc, Xue 2010; Xue et al. 2022). *Chenopodium* (fat hen) was also found in great quantities and associated with cereals remains, especially rice and millet grains, and it has been hypothesized as being cultivated (Dal Martello 2020; Xue et al. 2022). Several fruits and legumes were also found, including soybean (*Glycine* max), peaches (*Prunus persica*), apricots (*Prunus armeniaca*), raspberries (*Rubus* sp.), grapes (*Vitis* sp.), melons (*Cucumis* cf. *melo*) and jujube (*Ziziphus jujuba*). Over 800 cannabis grains were recovered in the archaeobotanical samples from Haimenkou. The majority of cannabis seeds (~ 700) were retrieved from a single context dated to 1400−1100 bc (Dal Martello 2020; Xue et al. 2022, Figs. 2 and 3). The cannabis seeds from Haimenkou were preserved by charring and have a slightly elongated shape with a smooth surface, and no pronounced basal caruncle.

**Fig. 2:**
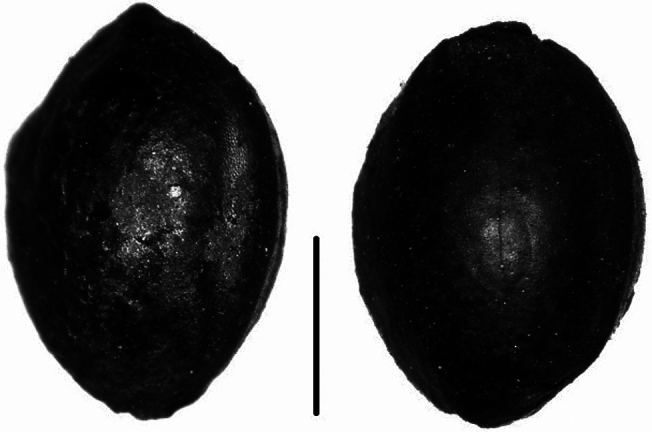
Photos of charred *Cannabis* sp. grains from Haimenkou, scale bar 2 mm

**Fig. 3:**
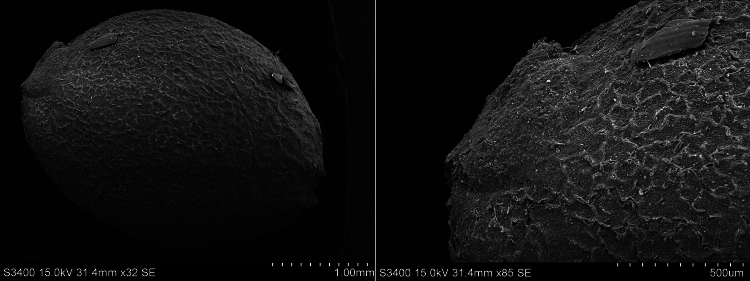
SEM photos of a *Cannabis* sp. seed from Haimenkou; right: close-up of hilum

### Metrics on cannabis achenes and collection of metrics

**Fig. 4:**
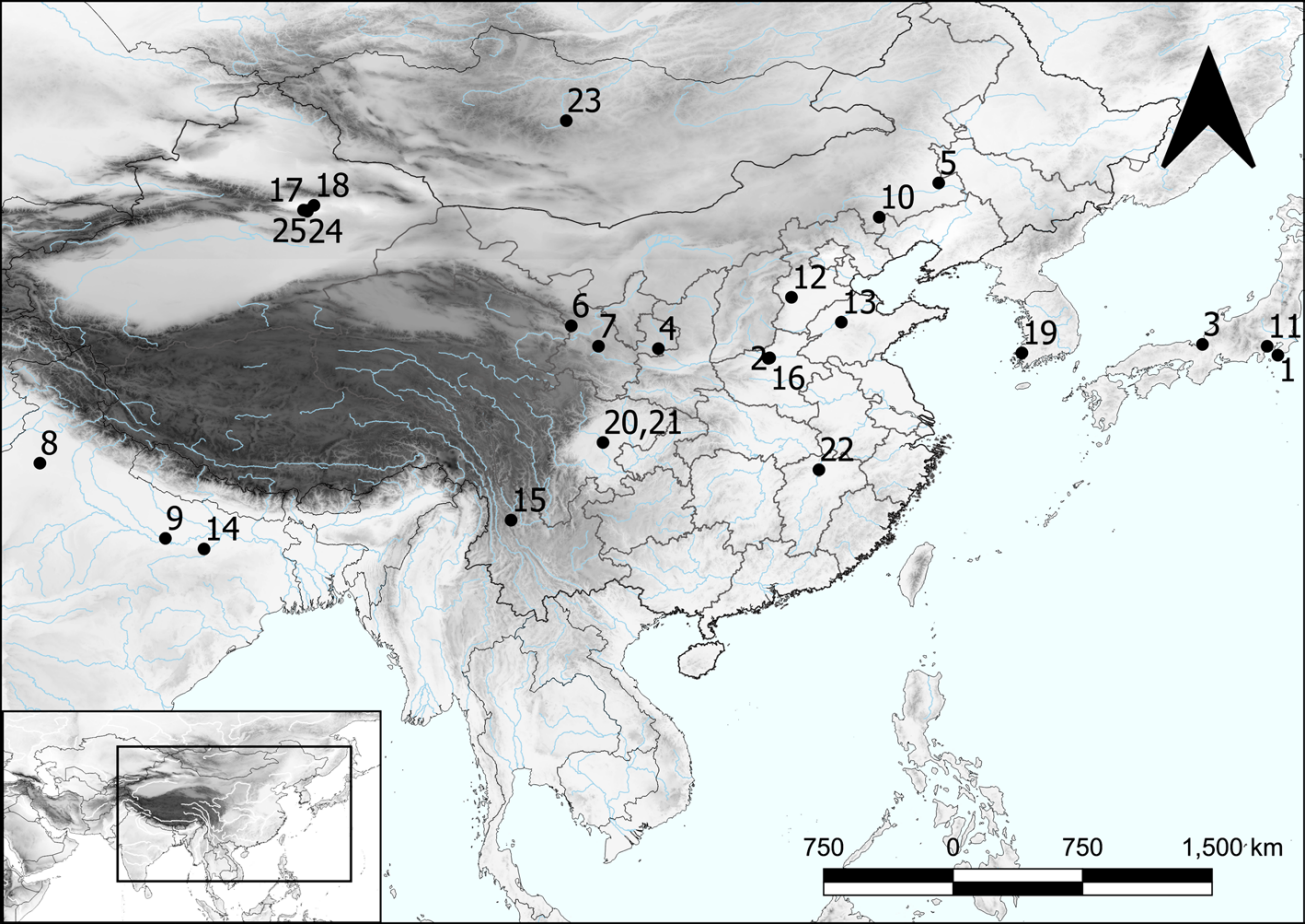
Location of Haimenkou and other sites mentioned in text: 1 Okinoshima; 2 Zhuzhai; 3 Torihama; 4 Yanggua; 5 Hamin Mangha; 6 Jinchankou; 7 Buziping; 8 Kunal; 9 Hetapatti; 10 Erdaojingzi; 11 Shimoyakebe; 12 Gaocheng Taixi; 13 Dazingzhuang; 14 Senuwar; 15 Haimenkou; 16 Guangzhuang; 17 Jiayi; 18 Yanghai; 19 Shinchangdong; 20 Laoguanshan M2; 21 Laoguanshan M3; 22 Marquis Haihun Graveyard; 23 Qara Qorum; 24 Karakhoja; 25 Astana. Made with QGIS

**Fig. 5:**
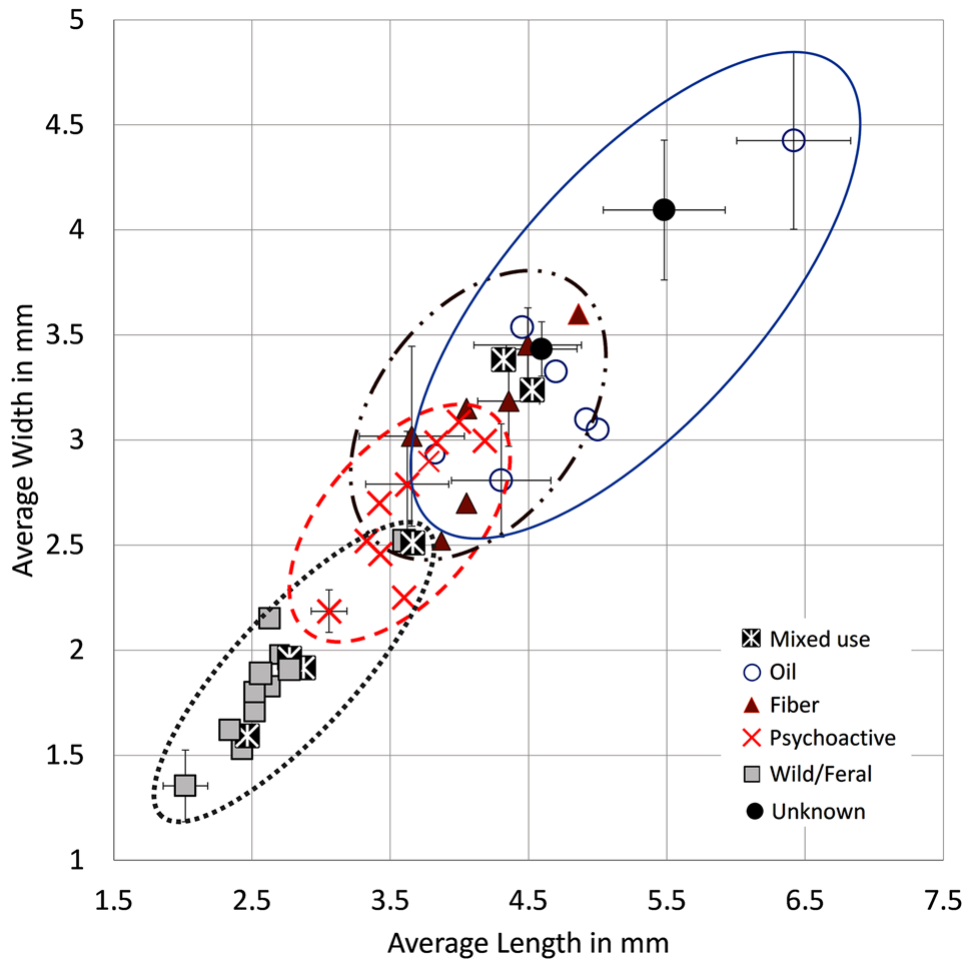
Comparison of modern cannabis achene measurements (shown corrected by -10%, see Table 2 and ESM 2 for original and corrected measurements; see ESM 1 Fig. S1 for indication of provenance; data from Emboden 1974; Small and Cronquist 1976; Russo 2007; Taheri-Garavand et al. 2012; Piluzza et al. 2013; Small 2015; Bouayoun et al. 2018; Asadi et al. 2019; McPartland and Small 2020; Moon et al. 2020; Kaliniewicz et al. 2021

**Fig. 6:**
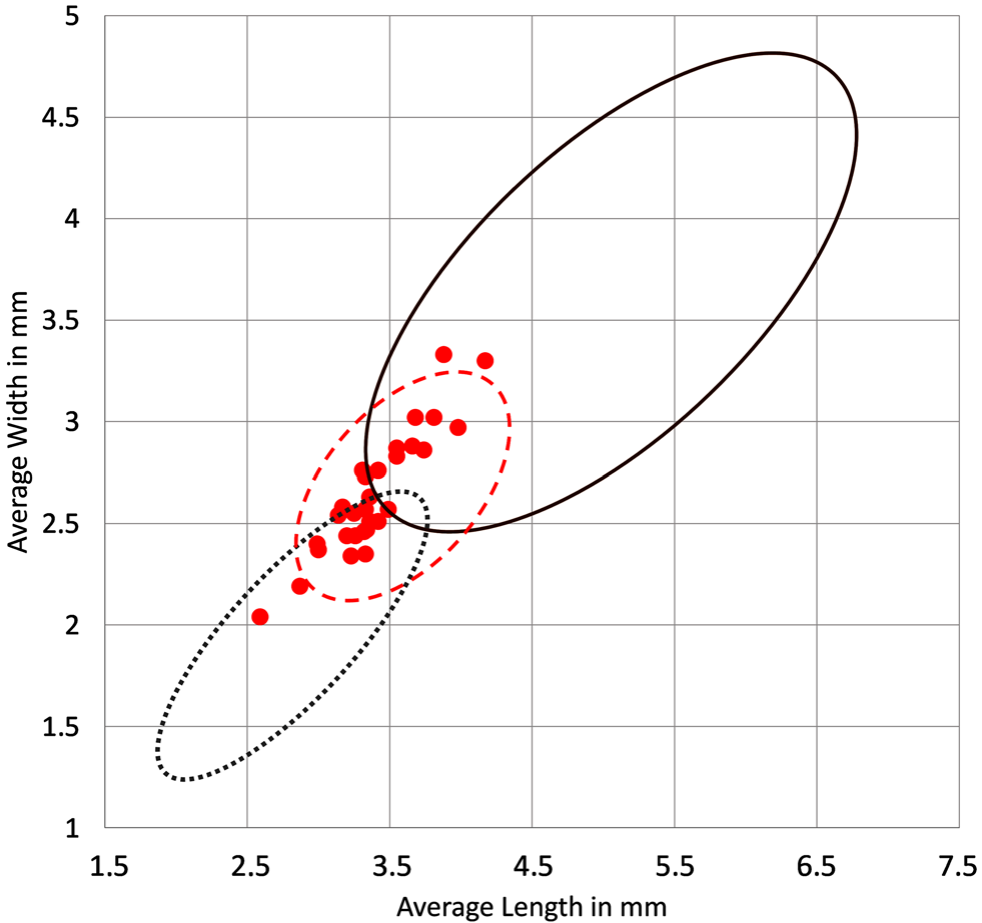
Scatterplot of L/W measurements from Haimenkou, circles showing modern cannabis seed size range corrected by -10% to account for the charring shrinking of archaeobotanical seeds as represented in this figure; fibre and oil ranges are grouped together

**Fig. 7:**
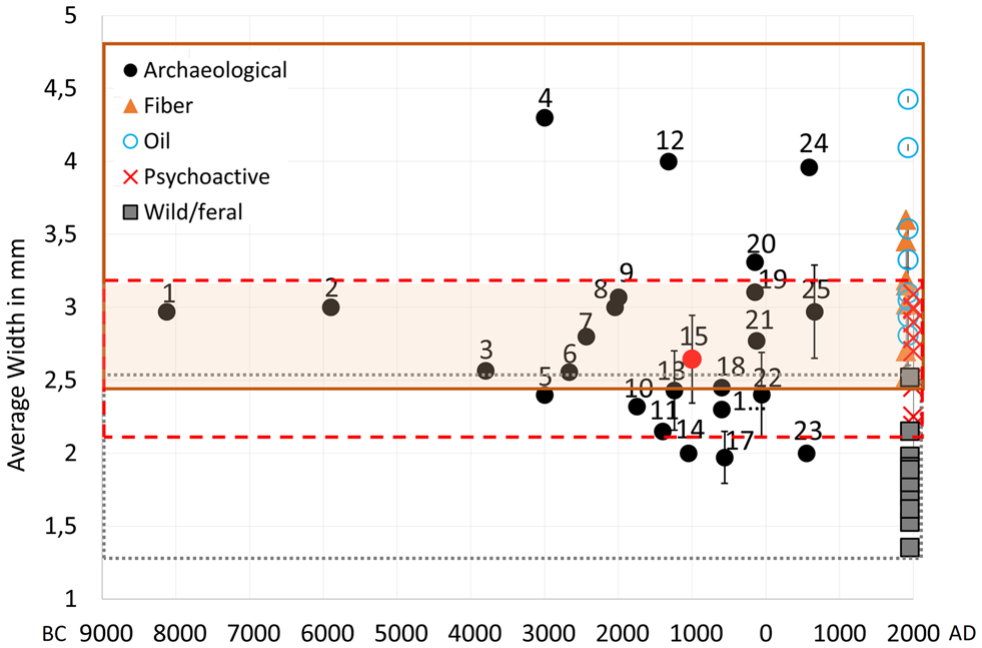
Average seed width of cannabis grains from archaeological sites: 1 Okinoshima; 2 Zhuzhai; 3 Torihama; 4 Yanggua; 5 Hamin Mangha; 6 Jinchankou; 7 Buziping; 8 Kunal; 9 Hetapatti; 10 Erdaojingzi; 11 Shimoyakebe; 12 Gaocheng Taixi; 13 Daxingzhuang; 14 Senuwar; 15 Haimenkou; 16 Guanzhuang; 17 Jiayi; 18 Yanghai; 19 Shinchangdong; 20 Laoguanshan M2; 21 Laoguanshan M3; 22 Marquis Haihun Graveyard; 23 Qara Qorum; 24 Karakhoja; 25 Astana. Charred and corrected values for desiccated, waterlogged and partially charred materials have been plotted (see Table 3 and ESM 2 for original and corrected values). Archaeological data from Kasahara 1987; Lee 2003; Saraswat and Pokharia 2003; Saraswat 2004; Rösch et al. 2005; Jiang et al. 2006; Chen 2007; Kobayashi et al. 2008; Zhou et al. 2011; Chen et al. 2012; Jia et al. 2013; Sun 2014; Yang 2014; Jiang et al. 2016; Pokharia et al. 2017; Bestel et al. 2018; Chen et al. 2019; Dal Martello 2020; Bai et al. 2021; Jiang et al. 2021

Thirty grains of cannabis were measured from the archaeobotanical samples of Haimenkou (see ESM 1 Table S2 for measurements on individual grains from Haimenkou); additionally, modern and archaeological measurements of cannabis achenes have been collected from published studies from locations across Eastern and South Asia (China, Japan, Korea and India); further measurements were obtained from published photos (Table 2 lists modern seeds metrics, Table 3 lists available archaeological seeds metrics, see ESM 2 for complete lists and references of both modern and archaeological datasets, see Fig. 4 for location of archaeological sites included in this study). Measurements on modern grains provide guidelines for distinguishing fibre and psychoactive varieties, and modern cannabis metrics have been collected from the available literature. In order to compare modern measurements with archaeological ones, we have applied a correction factor of -10% to account for the shrinkage caused by the charring process, in line with estimated correction factors applied to cereals and pulses when comparing charred vs. non-charred material (Hopf 1955; Hubbard 1976; Willcox 2004; Braadbaart and van Bergen 2005; Fuller and Harvey 2006; Braadbaart 2008; Märkle and Rösch 2008). Most of the archaeological cannabis seeds were preserved by charring; however, cannabis seeds from sites in Xinjiang, including Jiayi, Yanghai, Astana and Karakhoja, were preserved by desiccation; seeds from Torihama in Japan, Shinchangdong in Korea were preserved by waterlogging, and those found in Han Dynasty period graves from the Laoguanshan cemetery in Sichuan were reported as being partially charred. We have applied a correction factor of -10% to the desiccated/waterlogged seeds, in line with shrinkage factors obtained from experimental charring for cereal grains, and of -5% to the partially charred seeds, in order to account for the different preservation status and make the non-charred seeds comparable to charred ones (see Table 3 and ESM 2). We provide both original and corrected measurements in the tables below and in ESM 2, and for the purpose of our analyses, we plot charred and corrected values on the graphs below (Figs. 5, 6 and 7).

**Table 2:** List of modern cannabis measurements with indication of provenance, types according to original publications, average length and width, and averages corrected by -10%

	Provenance	Type in publication	Mean L (mm)	L-10%	Mean W (mm)	W-10%	References
1	Russia, Orel	Fibre	4.5	4.05	3.5	3.15	Emboden (1974)
2	Afghanistan	Fibre	5.4	4.86	4	3.6	Emboden (1974)
3	Unknown	Fibre	4.3	3.87	2.8	2.52	Small (2015)
4	Unknown	Fibre	4.061	3.655	3.354	3.018	Figure 8 A in Small (2015)
5	Netherlands	Fibre	4.994	4.494	3.836	3.452	Piluzza et al. (2013)
6	Romania	Fibre	4.5	4.05	3	2.7	Small and Cronquist (1976)
7	Korea	Fibre	4.84	4.356	3.54	3.186	Moon et al. (2020)
8	India, Delhi	Psychoactive	4	3.6	2.5	2.25	Emboden (1974)
9	Turkey, Izmir	Psychoactive	3.8	3.42	3	2.7	Emboden (1974)
10	Unknown	Psychoactive	3.398	3.058	2.428	2.185	Figure 8 A in Small (2015)
11	Netherlands	Psychoactive	4.025	3.622	3.1	2.79	Piluzza et al. (2013)
12	India	Psychoactive	3.7	3.33	2.8	2.52	Small and Cronquist (1976)
13	Afghanistan	Wild/feral	2.8	2.52	1.9	1.71	Emboden (1974)
14	Kafiristan	Wild/feral	3	2.7	2.2	1.98	Emboden (1974)
15	Russia, Saratov	Wild/feral	4	3.6	2.8	2.52	Emboden (1974)
16	Unknown	Wild/feral	2.92	2.628	2.03	1.827	Small (2015)
17	Kashmir	Wild/feral	2.243	2.018	1.504	1.354	Russo (2007) in Bestel et al. (2018)
18	Dzhungar, ex URSS	Wild/feral	2.8	2.52	2	1.8	Small and Cronquist (1976)
19	India	Wild/feral	2.7	2.43	1.7	1.53	Small and Cronquist (1976)
20	Netherlands	*Unspecified*	5.105	4.594	3.815	3.433	Piluzza et al. (2013)
21	China	Oil	7.130	6.417	4.916	4.424	Russo (2007) in Bestel et al. (2018)
22	Iran	Oil	4.249	3.824	3.26	2.934	Asadi et al. (2019)
23	Poland	Oil	4.78	4.302	3.12	2.808	Kaliniewicz et al. (2021)
24	Poland	*Unspecified*	6.09	5.481	4.55	4.095	Kaliniewicz et al. (2021)
25	Morocco	Oil	5.555	4.999	3.388	3.049	Bouayoun et al. (2018)
26	Morocco	Oil	5.22	4.698	3.695	3.325	Bouayoun et al. (2018)
27	Morocco	Oil	5.464	4.918	3.442	3.098	Bouayoun et al. (2018)
28	Iran	Oil	4.95	4.455	3.93	3.537	Taheri-Garavand et al. (2012)
29	Bangladesh	Psychoactive	4.2	3.78	3.22	2.898	McPartland and Small (2020)
30	India	Psychoactive	3.81	3.429	2.73	2.457	McPartland and Small (2020)
31	South Africa	Psychoactive	4	3.6	2.981	2.682	McPartland and Small (2020)
32	India	Mix use (psychoactive/fibre)	3.19	2.871	2.13	1.917	McPartland and Small (2020)
33	India	Mix use (psychoactive/fibre)	3.08	2.772	2.18	1.962	McPartland and Small (2020)
34	Bangladesh	Mix use (psychoactive/fibre)	2.74	2.466	1.77	1.593	McPartland and Small (2020)
35	Afghanistan	Mix use (psychoactive/oil)	4.07	3.663	2.79	2.511	McPartland and Small (2020)
36	Afghanistan	Mix use (psychoactive/oil)	4.8	4.32	3.76	3.384	McPartland and Small (2020)
37	China (Xinjiang)	Mix use (psychoactive/oil)	5.03	4.527	3.6	3.24	McPartland and Small (2020)
38	Kyrgystan	Wild/feral	2.92	2.628	2.39	2.151	McPartland and Small (2020)
39	Afghanistan	Wild/feral	2.6	2.34	1.8	1.62	McPartland and Small (2020)
40	Afghanistan	Wild/feral	3.08	2.772	2.12	1.908	McPartland and Small (2020)
41	Kyrgystan	Wild/feral	2.85	2.565	2.1	1.89	McPartland and Small (2020)
42	U.S.A.	Psychoactive	4.26	3.834	3.32	2.988	McPartland and Small (2020)
43	Lebanon	Psychoactive	4.44	3.996	3.43	3.087	McPartland and Small (2020)
44	Lebanon	Psychoactive	4.65	4.185	3.33	2.997	McPartland and Small (2020)

Modern metrics plotted in Fig. 5 are corrected by -10%

**Table 3:** List of archaeological cannabis measurements with indication of provenance, site location and chronology

	Site	Preservation status	Chronology (bc)	Mean L (mm)	Corr. L	Mean W (mm)	Corr. W	References
1	Okinoshima	Waterlogged	8260 − 7990	3.6	3.24	3.3	2.97	Kobayashi et al. (2008)
2	Zhuzhai	Charred	5974 − 5823	3.6		3		Bestel et al. (2018)
3	Torihama	Waterlogged	4200 − 3400	3.25	2.925	2.85	2.565	Kasahara (1987)
4	Yangua	Charred	3000	5.1		4.3		Zhou et al. (2011)
5	Hamin Mangha	Charred	3000	3.3		2.4		Sun (2014)
6	Jinchankou	Charred	2670	3.12		2.556		Yang (2014)
7	Buziping	Charred	2940 − 1930	3.72		2.8		Jia et al. (2013)
8	Kunal	Charred	2600 − 1500	5		3		Saraswat and Pokharia (2003)
9	Hetapatti	Charred	2500 − 1500	3.54		3.07		Pokharia et al. (2017)
10	Erdaojingzi	Charred	2000 − 1500	3.26		2.32		Sun (2014)
11	Shimoyakebe	Charred	1400	3.2		2.15		Sasaki et al. (2007); Crawford 2011)
12	Gaocheng Taixi	Charred	1600 − 1046	4.5		4		Chen (2007)
13	Daxingzhuang	Charred	1435 − 1050	3.168		2.432		Chen (2007)
14	Senuwar	Charred	1400 − 700	2.45		2		Saraswat (2004)
15	Haimenkou	Charred	1600 − 400	3.391		2.643		Dal Martello (2020)
16	Guanzhuang	Charred	800 − 400	3.2		2.3		Tang et al. (2022)
17	Jiayi	Desiccated	800 − 400	3.792	3.413	2.725	2.452	Jiang et al. (2016)
18	Yanghai	Desiccated	820 − 300	2.99	2.691	2.19	1.971	Jiang et al. (2006)
19	Shinchangdong	Waterlogged	200 − 100	4.25	3.825	3.45	3.105	Lee (2003)
20	Laoguanshan M2	Partially charred	157 − 141	4.16		3.31		Bai et al. (2021)
21	Laoguanshan M3	Partially charred	157 − 141	3.46		2.77		Bai et al. (2021)
22	Marquis Haihun Graveyard	Waterlogged	59	3.275	2.948	2.668	2.401	Jiang et al. (2021)
23	Qara Qorum	Charred	ad 450–750	2.9		2		Rosch et al. (2005)
24	Karakhoja	Desiccated	ad 265–907					Chen et al. (2019)
25	Astana (Xinjiang)	Desiccated	ad 658	4.23	3.807	3.3	2.97	Chen et al. (2012)

Corrected values are signified for desiccated and waterlogged (-10%) and partially charred (-5%) material

## Results

### Modern metrics on cannabis grains

Modern cannabis seeds show distinct sizes for psychoactive and fibre type cannabis (Small 2015, Fig. 5). According to Small and Cronquist’s (1976) early work on cannabis type differentiation, seeds of domesticated cannabis fibre varieties have a length of at least 3.8 mm, with shorter seeds belonging to wild/feral and psychoactive types. Our collection of modern published measurements shows that wild/feral cannabis seeds, which include *spontanea* and *asperrima*/*kafiristanica* varieties, range between 2.24 and 4 mm in length (-10% values: 2.01–3.06 mm), and 1.5–2.8 mm in width (-10% values: 1.35–2.52 mm). Modern psychoactive cannabis seeds range between 3.3 and 4.6 mm in length (-10% values: 3.05–4.1 mm), and 2.4–3.4 mm in width (-10% values: 2.18–3.08). Modern fibre cannabis seeds range between 4.06 and 5.4 mm in length (-10% values: 3.65–4.86 mm) and 2.8−4 mm in width (-10% values: 2.52–3.6 mm), and oil-type cannabis seeds range between 4.2 and 7.1 mm in length (-10% values 3.8–6.4 mm) and between 3.12 and 4.42 mm in width (-10% values 2.8–4.4 mm; Table 2 and ESM 2). Measurements provided in Table 2 comprise modern metrics, both original and corrected by -10% values. Following methods of several recorded crop domestication studies based on grain metrics that showed width is the most affected dimension during the initial domestication phase (e.g. Fuller et al. 2014, 2017, 2019, 2021), we have chosen to plot width for both modern and archaeological seeds in our analyses below; we plot corrected values to allow for comparison with archaeologically charred material. From modern measurements, we consider width ranging between 2.4 and 3.1 mm (-10% corrected value) as the overlapping range between psychoactive and fibre cannabis; width below 2.4 mm (-10% corrected value) as distinctive of psychoactive cannabis, and width above 3.1 mm (-10% corrected value) as distinctive of fibre and oil cannabis. Since fibre and oil type cannabis seed size ranges largely overlap, with all fibre accession falling within the range of oil accessions (Fig. 5), and the two are conventionally recognized as the same subspecies, below we grouped them in the category fibre/oil (Table 2; Figs. 6 and 7).

### Haimenkou cannabis grain metrics

The cannabis grains from Haimenkou measured on average 3.39 mm in length, 2.2 mm in width, and 1.2 mm in thickness (see Table 3; Fig. 6; ESM 1 Table S2 and ESM 2). A scatterplot of the measurements from Haimenkou shows that the majority of the seeds plot in the overlapping area between fibre/oil and psychoactive cannabis, according to our collection of modern cannabis seeds metrics. A large number, just over 50%, fall within the expected distribution of wild/feral types, but the absence of a caruncle (Fig. 2) argues against this. While the majority fall within the distribution of psychoactive type, about half fall within the fibre/oil type range with a few grains showing size comparable to only fibre types. We therefore suggest that these represent an early form of ssp. *chinensis* or ssp. *indica* before larger seeded oilseed forms had been selected or were available in the region. The archaeological contexts from which cannabis seeds have been retrieved at Haimenkou suggest storage as a food grain, i.e. as oilseed use. This derives from the observation that a single sample, hand collected, contained more than 700 cannabis seeds, thus suggesting charring of a cluster of seeds from part of a stored unit rather than a mixed context. Similar examples of charred samples consisting of almost exclusively clean food grains from the site, included samples with thousands of foxtail millet grains (*Setaria italica*) and another consisting of rice grains and the pseudo-cereal *Chenopodium* cf. *album* (Xue et al. 2022).

#### Archaeological cannabis grain metrics

Published measurements on archaeological cannabis achenes from sites in East and South Asia have been collected and compared with those obtained from Haimenkou (Table 3; Figs. 5 and 7). Table 3 provides archaeological metrics, including corrected values for desiccated/waterlogged and partially charred archaeological seeds, -10% and -5%, respectively. Average width has been plotted against chronology (median), following methods for tracking grain size change used across many other crops (e.g. Fuller and Allaby 2009; Purugganan and Fuller 2011). This shows that the earliest available reported cannabis grains with metrics (pre-3000 bc) plot within the overlapping area of psychoactive/fibre cannabis. Since even the earliest finds at Okinoshima, Japan (cannabis seeds directly dated) lack a wild-type caruncle, it can be suggested that all of these early records are likely to represent cultivated plants (for definitions of cultivated vs. domesticated plants please see Fuller and Hildebrand 2013). The smaller size is consistent with an early domesticate, but is larger than the expected wild range. Width of grains shows differentiation towards wider grains from ca. 3000 bc onward, suggestive of specialized oilseed varieties. These large seeded examples occur in Xinjiang, represented by the desiccated material found at Yangua, Xinjiang (ca. 3000 bc, Zhou et al. 2011), however Yanghua metrics fall well above modern metrics and might not be totally reliable. Other larger, possible fibre records include Hetapatti (ca. 2000 bc, Pokharia et al. 2017) in India; Gaocheng Taixi (ca. 1600−1046 bc, Chen 2007), and Haimenkou (ca. 1600−400 bc, this study, Xue et al. 2022) in China. It remains to be resolved whether Indian occurrences pre-date 2000 bc (Fuller and Murphy 2018), but this nevertheless implies selection for fibre and oilseed uses began prior than 1500 bc.

**Fig. 8:**
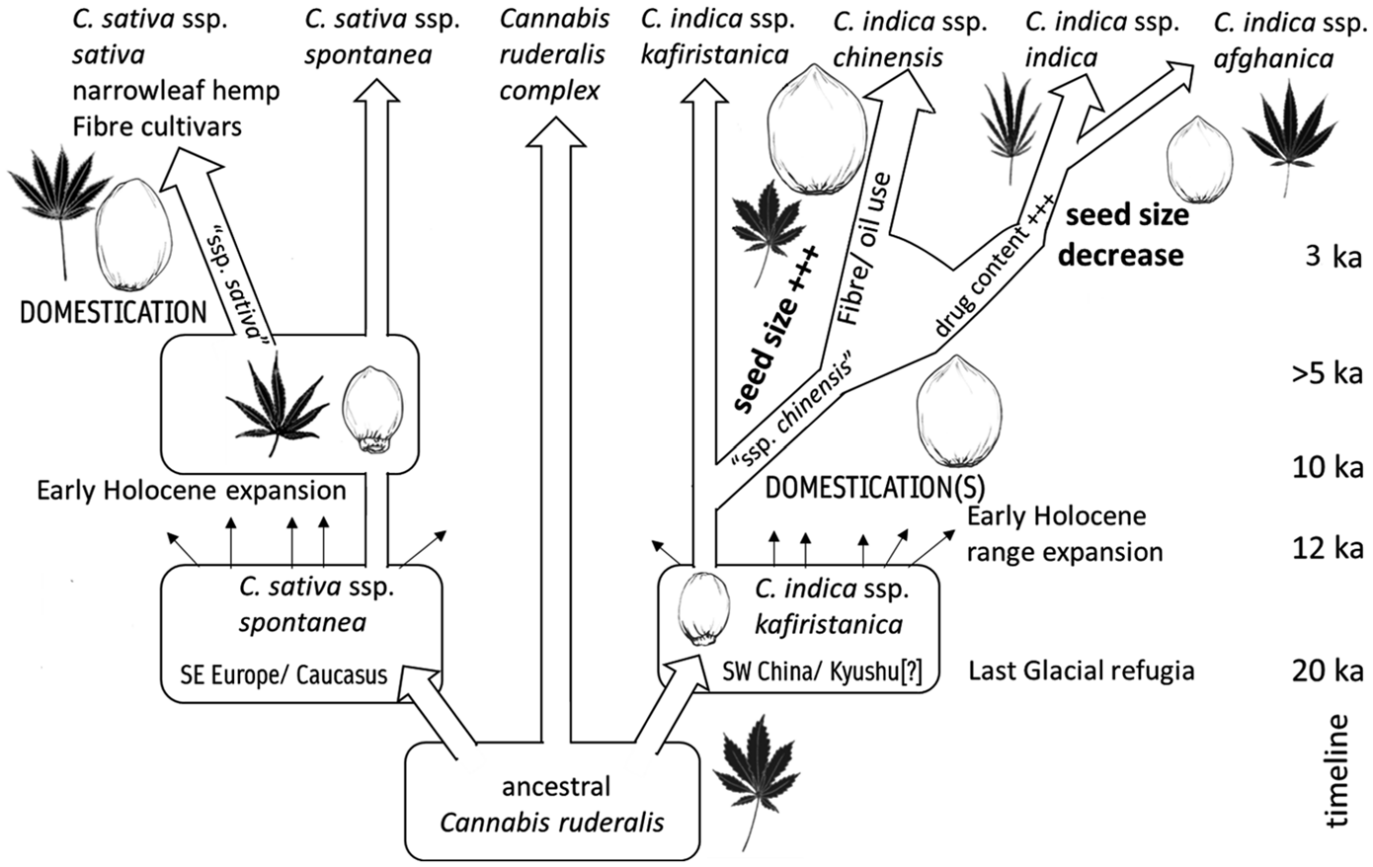
Diagram showing suggested evolution of cannabis, with proposed timeline (approximate in 1,000s of years bp [kya]) of phylum divergence and range expansion events in eastern Eurasia in light of archaeological evidence presented in this paper. For example, post-glacial expansion and radiation takes place between 20,000 and 12,000 bp, domestication episodes take place between 10,000 and 5,000 bp, and special use subspecies are established variously before or after 3,000 bp

A second evolutionary trajectory can be suggested as beginning ca. 2000 bc with a trend towards smaller seeded populations, suggested as including the specialized psychoactive varieties of ssp. *indica*, perhaps selected for higher THC content, although some feral populations could also be included (Fig. 8). This is represented by finds from the following: Hamin Mangha (ca. 3000 bc, Sun 2014), Erdaojingzi (ca. 2000−1500 bc, Sun 2014) and Yanghai, China (820−300 bc, Jiang et al. 2006); Shimoyakebe, Korea (ca. 3,400 bp, Sasaki et al. 2007; Crawford 2011); Senuwar, India (1400−700 bc, Saraswat 2004). The examples from Yanghai, Shimoyakebe and Senuwar are clearly illustrated and lack a caruncle, indicating these are small-seeded domesticates.

## Discussion

Although still limited, the available morphometric data shows that all of the archaeological cannabis grain size reports collected for this study are comparable to known grain size in varieties of modern cultivated cannabis. This is consistent with the hypothesis of an early exploitation of the plant. Seeds coming from sites dating from ~ 8000 to ~ 3000 years bc fall in the grey area of undifferentiated cultivars; this would suggest they fit within a broader conception of *C. sativa* ssp. *indica*, which plausibly has variable THC content and could indeed have been used for psychoactive purposes (Fig. 8), as argued by Clarke and Merlin (2013). Nevertheless, a generalized use, including some for edible seeds and fibre use is plausible, and food uses are more likely to have resulted in archaeological preservation.

Some of the earliest finds of cannabis in the world come from the site of Okinoshima, Japan; these have been directly dated to around 8000 bc (8280−7660 cal bc, NUTA2-12809, Kobayashi et al. 2008; Kudo et al. 2009). The widths of these seeds fall outside the size range of wild cannabis, and lack the caruncle of var. *asperrima*/*kafiristanica*, thus we can infer that these are likely already a domesticated form of ssp. *indica.* Similarly to Okinoshima seeds, cannabis seeds reported from another Jomon site, Torihama (ca. 5000 bc, Kudo et al. 2009) also, from the photographs, lack a caruncle and no prominent abscission scar is evident (cf. online resource 1 Table S1 in McPartland and Hegman 2018). Whilst the find was interpreted as evidence for introduction and cultivation in Japan (Kobayashi et al. 2008), the early date would suggest that potentially it represents one of the earliest known East Asian domesticates. In China, the earliest reported grains come from Zhuzhai (ca. 5900−5800 bc, Bestel et al. 2018), and their size is greater than known wild seeds, suggesting possible cultivation of cannabis in the Middle Yellow River region of China at an early date. Since there is no evidence for contact between Japan and China at this time for any dispersal of crops (e.g. rice, millets, azuki bean and soybean all only appear to disperse across these regions after 3500 bc, Stevens and Fuller 2017), cultivation of cannabis plausibly had begun independently in at least China and Japan.

Use for fibre and for edible seeds would have been pre-requisites for selection for specialized varieties within ssp. *indica* var. *chinensis*, which evolved larger seeds, with the largest found in those varieties specialized for oilseed use. Archaeologically, such large grains have been reported from at least 4000 bc in China (e.g. Yanghua, Gaocheng Taixi), and from perhaps 2000 bc in India (e.g. Hetapatti). This implies that by the 2nd millennium bc, differentiation of hemp for fibre and/or oil seed varieties (ssp. *chinensis*) from wild varieties had taken place across broader East and South Asia. The selection process for larger seeds is unclear. One possibility is a phase of competitive selection (*sensu* Allaby et al. 2022), brought about by denser planting or more intensive field preparation, including manuring. Denser planting could also drive selection for taller plants, which came to characterize fibre varieties. It is also possible that larger seeds were brought on by allometric links to larger overall plant size, suggested as playing a role in some domestication processes (Milla and Matesanz 2017).

Early claims of hemp textiles from archaeological sites in China have later been disputed, being mostly based on fabric impressions on ceramics (Bergfjord and Holst 2010; Haugan and Holst 2013). In addition to seeds, securely identified hemp fibre remains come from Gaocheng Taixi, in Hebei, where a complete roll of woven hemp has been recovered (Shang Dynasty, ca. 1600−1046 bc, Cameron 2010). A roll of hemp fabric was also recovered from tomb no. 1 at Mawangdui near Changsha (ca. 200 bc–ad 200, Cheng 1992), and hemp was also reported from Kwo La Wan in Hong Kong (1300−1000 bc, Meacham 1994, pp 184–185). Historical records also show that hemp cloth was used as tax payment during the Zhou dynasty (1045−256 bc), together with grains (Kuhn 1988). At Haimenkou, cannabis seed size mostly plots in the range of overlapping psychoactive/fibre types (Fig. 6); we therefore suggest that the cannabis assemblage from Haimenkou is indicative of a crop beginning to undergo evolution from its early domesticated form towards a diversified crop with multiple uses, including larger oilseed/ fibre adapted varieties. These can probably be attributed to ssp. *indica* var. *chinensis*.

In mainland Southeast Asia, at the site of Ban don Ta Phet in central Thailand (ca. 400 bc), hemp has been identified among the numerous textile fragments, and it has been suggested that it was an exotic material coming from China (Cameron 2010). Hemp fibres have also been reported used as clay plaster mixture in the Ellora Cave in India, dating to ca. 6th–11th centuries ad (Singh and Saresdai 2016).

Smaller cannabis seeds appear from ca. 3000 bc, e.g. at Hamin Mangha (China), Shimoyakebe (Japan), and Senuwar (India). While in some cases these might be of wild plants, at least from Jiayi and Yanghai, where seeds have been recovered in association with female plants in burial contexts, suggest it was possibly a medicinal/psychoactive variety (see below).

As seen from the written Chinese records, differentiation of cannabis for fibre/oil use from psychoactive use varieties was coming into existence at least by the first millennium bc. In Indian written sources, early use of the fibre is recorded in Indian languages such as Pali, Prakrit and later Sanskrit, placing it from the first millennium bc through early centuries ad. The Late Vedic Sanskrit (also Pali) *śaṇa* indicates fibre hemp (Rhys-Davids and Stede 1925; Turner 1966), while Prakrit *ganjā* is psychoactive hemp (Turner 1966). In addition, *bhaṅgá* in Pali and later Vedic Sanskrit has glosses for both drug use and fibre use (Rhys Davids and Stede 1925; Turner 1966), which may indicate the persistence of mixed purpose crops. Hymns of the Atharaveda (ca. 1000 bc) also list *bhanga* alongside other drugs such as *soma* (Russo 2005). While we have posited psychoactive use since the Early Holocene (as hypothesized by Clarke and Merlin 2013), the higher THC varieties with smaller seeds had evolved by the 2nd millennium bc and 1st millennium bc when they are found in India.

In addition to seeds whole female plants with inflorescence have been reported from graves at the Jiayi and the slightly later Yanghai sites in Xinjiang (Jiang et al. 2006, 2016); these have been interpreted as clear indication of a ritualistic use of the plant (Jiang et al. 2016). At the Han Dynasty period Laoguanshan graves in Sichuan, Southwest China, thousands of seeds have been reported in tombs M3 and M2 (Bai et al. 2021). In tomb M2 in addition to cannabis grains there were four models of weaving machines, suggesting that the deceased buried in M2 was involved in textile production. In Tomb M3 a mortar and pestle and bamboo strips with medical recipes were part of the burial goods, suggesting that the deceased male buried there was a doctor, and further indicating a medicinal use of cannabis by the late 1st millennium bc in China (Bai et al. 2021).

The introduction of cultivated cannabis into India could have possibly derived from Central Asia through the Middle Asian crop exchange (Stevens et al. 2016). The evidence from Yanghai in Xinjiang might suggest that the dispersed variety could have been the drug form. Dispersal into South Asia from Central Asia for cannabis drug cultivars is also inferred on linguistic grounds, as argued on the basis of occurrences of cognate terms in Iranian and Indic languages, implying loans into Indo-Iranian, which Witzel (1999, 2005), Southworth (2005) and Southworth and McAlpin (2014) suggest may have been from a lost Central Asia language: this includes the terms *śaṇa* and *bhaṅgá (*Persian *šan* and *bhanga*). Witzel (2005) also suggests an original central Asian source *k’an, which would also evolve into *ganja*, Kirgiz *kandir*, Old Russian Church Slavic *konoplja*, and Greek *kánnabis.* In other words, Middle to Late Bronze Age central Asia would have served as a hub for the diffusion of cannabis varieties, together with their names.

## Conclusions

Although limited in scope, this study shows the potential of using grain morphometrics as an aid to disentangle the domestication trajectory and past use of cannabis. Through the collection of modern metrics, we have established a baseline for distinguishing fibre/oil and psychoactive/ritualistic cannabis seeds. This baseline provides a framework within which to analyze archaeological cannabis grains. This, together with a contextual analysis of the archaeological context in which seeds have been found, can provide insights into a more precise, unbiased interpretation of cannabis seeds remains from archaeological sites. According to the presently available data, cannabis exploitation has a long antiquity, with the earliest archaeological seeds already showing sizes comparable to modern varieties, differentiated for fibre or psychoactive use. An initial differentiation between fibre and psychoactive cannabis is detected from ca. 3000 bc for fibre cannabis, and derived oilseed varieties of ssp. *chinensis.* From ca. 2000 bc, smaller seeded, and presumably high THC psychoactive varieties evolved, and they are present in East Asia and South Asia. Future work should focus on collecting a wider range of measurements.

### Supplementary Information

Below is the link to the electronic supplementary material.

Supplementary material 1 (PDF 212 kb)

Supplementary material 2 (PDF 125 kb)

Supplementary material 3 (PDF 411 kb)

Supplementary material 4 (XLSX 26 kb)

## Data Availability

All data provided is available online as ESM.
