# Supplementary material for: Morphometric approaches to Cannabis evolution and differentiation from archaeological sites: interpreting the archaeobotanical evidence from bronze age Haimenkou, Yunnan
Source: Veg Hist Archaeobot. 2023 Nov 30;33(4):503–18. doi: 10.1007/s00334-023-00966-6 (PMC11127845; doi:10.1007/s00334-023-00966-6)
Supplement: Supplementary file 1 — Supplementary material 1 (PDF 212 kb) [file 334_2023_966_MOESM1_ESM.pdf]

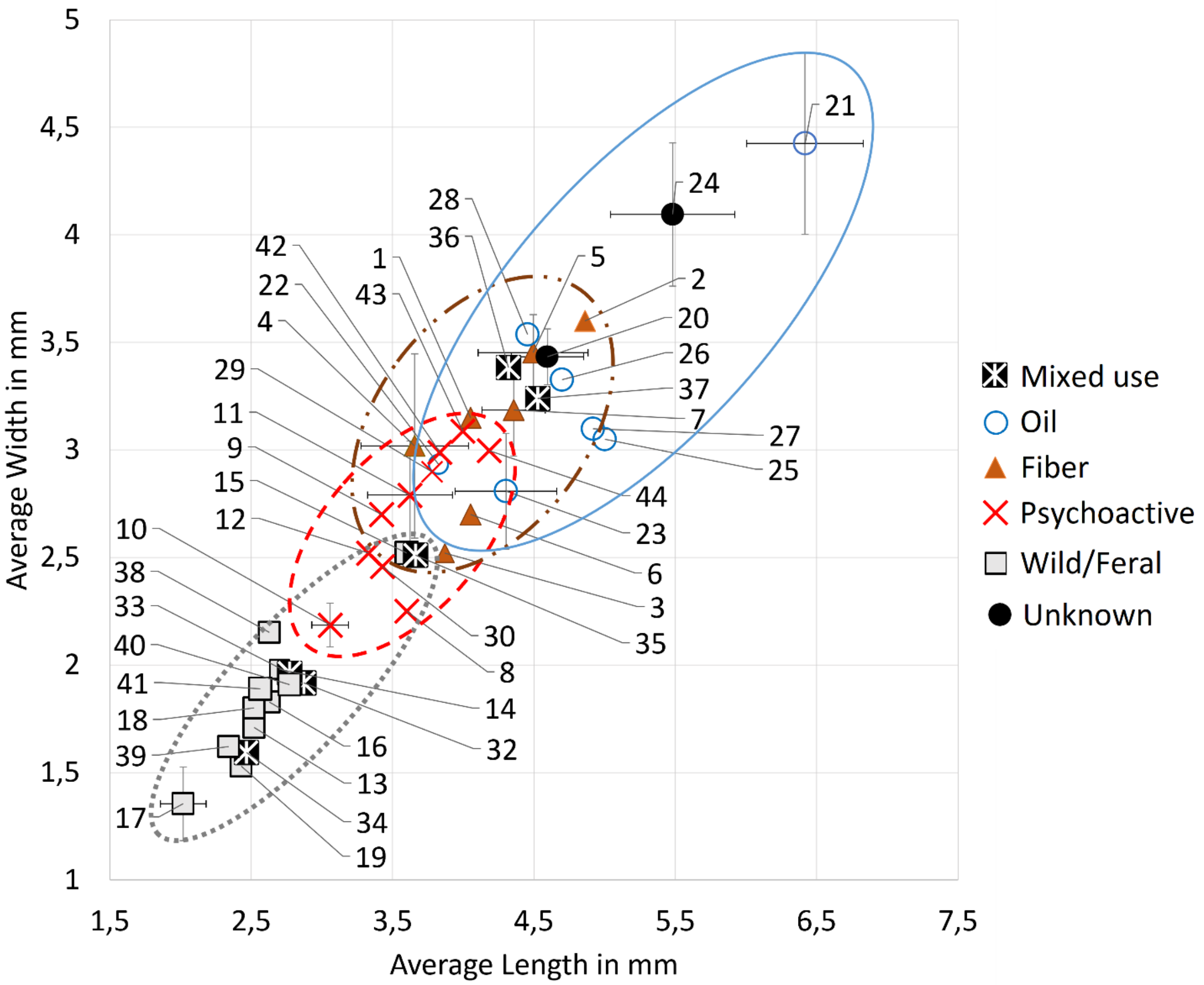

ESM1 Fig. S1. Modern cannabis achene measurements (shown corrected by -10%, see Table 2 in text and Supplementary Table S3 for original and corrected measurements). Seeds from: 1. Russia, Orel; 2. Afghanistan; 3. Unknown; 4. Unknown; 5. Netherlands; 6. Romania; 7. Korea; 8. India, Delhi; 9. Turkey, Izmir; 10. Unknown; 11. Netherlands; 12. India; 13. Afghanistan; 14. Kafiristan; 15. Russia, Saratov; 16. Unknown; 17. Kashmir; 18. Dzhugar; 19. India; 20. Netherlands; 21. China; 22. Iran; 23-24. Poland; 25-27 Morocco; 28. Iran; 29. Bangladesh, Rajshahi; 30. India, Combaitore; 31. South Africa; 32 India, Himachal Pradesh; 33. India, Bareilly; 34. Bangladesh, East Bengal; 35. Afghanistan, Ghazni Prov.; 36. Afghanistan, Kandahar Prov.; 37. China, Xinjiang, Yarkant; 38. Kyrgystan, Issyk-Kul Region; 39. Afghanistan, Nuristan; 40. Kazakhstan, Kailiyskiy Alatau; 41. Kyrgystan, Issyk-Kul Region; 42. U.S.A., California; 43-44. Lebanon. (Data from Emboden 1974; Russo 2007; Small 2015; Piluzza et al. 2013; Moon et al. 2020; Small and Cronquist 1976; Asadi et al. 2019; Kaliniewicz et al. 2021; Bouayoun et al. 2018; Taheri-Garavand et al. 2012; McPartland and Small 2020).
