# Supplementary material for: Morphometric approaches to Cannabis evolution and differentiation from archaeological sites: interpreting the archaeobotanical evidence from bronze age Haimenkou, Yunnan
Source: Veg Hist Archaeobot. 2023 Nov 30;33(4):503–18. doi: 10.1007/s00334-023-00966-6 (PMC11127845; doi:10.1007/s00334-023-00966-6)
Supplement: Supplementary file 2 — Supplementary material 2 (PDF 125 kb) [file 334_2023_966_MOESM2_ESM.pdf]

EMS1 Table S2.

Measurements of cannabis seeds from Haimenkou, Yunnan. All measurements in mm (from Dal Martello 2020).

Grain no.	Site	Context	Period	Length	Width	Thickness	L/W
1	2008 JHDT	1204-6	2	3.55	2.83	2.22	1.254417
2	2008 JHDT	1204-6	2	3.88	3.33	2.8	1.165165
3	2008 JHDT	1204-6	2	3.31	2.76	2.27	1.199275
4	2008 JHDT	1204-6	2	3.17	2.58	2.33	1.228682
5	2008 JHDT	1204-6	2	3.81	3.02	2.49	1.261589
6	2008 JHDT	1204-6	2	3.49	2.57	2.25	1.357977
7	2008 JHDT	1204-6	2	3	2.37	2.06	1.265823
8	2008 JHDT	1204-6	2	3.32	2.46	2.16	1.349594
9	2008 JHDT	1204-6	2	3.26	2.44	2.11	1.336066
10	2008 JHDT	1204-6	2	3.2	2.44	2.04	1.311475
11	2008 JHDT	1204-6	2	3.66	2.88	2.51	1.270833
12	2008 JHDT	1204-6	2	3.68	3.02	2.53	1.218543
13	2008 JHDT	1204-6	2	3.42	2.76	2.19	1.23913
14	2008 JHDT	1204-6	2	3.34	2.47	2.14	1.352227
15	2008 JHDT	1204-6	2	3.98	2.97	2.53	1.340067
16	2008 JHDT	1204-6	2	2.87	2.19	1.7	1.310502
17	2008 JHDT	1204-6	2	3.33	2.35	1.99	1.417021
18	2008 JHDT	1204-6	2	3.23	2.34	1.92	1.380342
19	2008 JHDT	1204-6	2	3.74	2.86	2.4	1.307692
20	2008 JHDT	1204-6	2	2.99	2.4	1.98	1.245833
21	2008 JHDT	1204-6	2	3.25	2.55	2.27	1.27451
22	2008 JHDT	1204-6	2	3.36	2.63	2.07	1.277567
23	2008 JHDT	1204-6	2	3.36	2.51	1.96	1.338645
24	2008 JHDT	1204-6	2	3.42	2.51	1.76	1.36255
25	2008 JHDT	1204-6	2	3.55	2.87	2.23	1.236934
26	2008 JHDT	1204-6	2	3.33	2.73	2.45	1.21978
27	2008 JHDT	1204-6	2	2.59	2.04	1.68	1.269608
28	2008 JHDT	1204-6	2	3.14	2.54	2.15	1.23622
29	2008 JHDT	1204-6	2	3.33	2.57	2.12	1.29572
30	2008 JHDT	1204-6	2	4.17	3.3	2.8	1.263636
**AVERAGE**				**3.391**	**2.643**	**2.203667**	**1.286248**
**STDEV**				**0.330802**	**0.300277**	**0.28024**	**0.059246**
