# Supplementary material for: Morphometric approaches to Cannabis evolution and differentiation from archaeological sites: interpreting the archaeobotanical evidence from bronze age Haimenkou, Yunnan
Source: Veg Hist Archaeobot. 2023 Nov 30;33(4):503–18. doi: 10.1007/s00334-023-00966-6 (PMC11127845; doi:10.1007/s00334-023-00966-6)
Supplement: Supplementary file 3 — Supplementary material 3 (PDF 411 kb) [file 334_2023_966_MOESM3_ESM.pdf]

ESM1 Table S1.

Quotes and translations of early written accounts of cannabis use from Chinese texts.

Historical text	Quote	Translation	Inferred use	References
**詩經 *Shī Jīng*<br>Book of Odes<br>c. 1000-700 BC**	蓺麻如之何、衡從其畝<br>[國風 - 齊風]	How do we proceed in planting hemp? The acres must be dressed lengthwise and crosswise.<br>[Lessons from the States: Odes of Qi]	Grain	Legge 1876; 1879<br>Li 1974<br>Huang 2000
	東門之池、可以漚麻<br>[國風 -陳風]	The moat at the East Gate is fit to steep hemp in.<br>[Lessons from the States: Odes of Chen]		
	九月築場圃、十月納禾稼。<br>黍稷重穋、禾麻菽麥。<br>[國風 -豳風]	In the ninth month we make ready the stackyards, in the tenth month we bring in the harvest.<br>Millet for wine, millet for cooking, the early and the late, paddy and hemp, beans and wheat.<br>[Lessons from the States: Odes of Bin]		
	誕實匍匐、克歧克嶷、以就口食。<br>蓺之荏菽、荏菽旆旆、禾役穟穟、麻麥幪幪、瓜瓞唪唪<br>[大雅 - 生民之什]	When he was able to crawl, He looked majestic and intelligent. When he was able to feed himself, He fell to planting beans. The beans grew luxuriantly; His rows of paddy shot up beautifully; His hemp and wheat grew strong and close; His gourds yielded abundantly.<br>[Greater Odes of the Kingdom: The Second Decade, or that of Sheng Min]		
**周禮 *Zhōu Lǐ*<br>the Rites of Zhou<br>c. 1000-700 BC<br>(written 300-100 BC)**	[⋯]治絲麻以成之，謂之婦功<br>[冬官考工記]	Travailler la soie, le chanvre, pour les perfectionner, c'est l'office des femmies ovriéres<br>[ou mémoire sur l'examen des ouvriers/ Offices of winter]	Fiber, growing and processing hemp was the duty of the female employees	Biot 1851<br>Li 1974: 443
	河南曰豫州，其山鎮曰華山，其澤藪曰圃田，其川熒、雒，其浸波、溠，其利林、漆、絲、枲，其民二男三女，其畜宜六擾，其穀宜五種	Les neuf espèces de grains sont: le millet *Chou (milium glo-bosum?),* le millet *Tsi (holcus sorghum*), le riz en général *Tao*, le riz qui produit une liqueur fermentescible; le chanvre, le grand *Teou (dolichos*), le petit *Teou (pois*), le grand *Me* (l'orge), le petit *Me* (le blé)<br>[Ministére de l'été, ou du pouvoir exécutif/	Mentioned as one of the nine *gu* in the commentaries (*gu* means grain, used to indicate both cereal and non-cereal grains)	

ESM1 Table S1, continued

	[夏官司馬]	Offices of summer]		
尚書 Shàng Shū **Book of Historical Documents 772 BC - 476 BC** <br>***Part 3: 夏書 Xià Shū*** **the book of Xia** ***1. 禹貢 Yǔ Gòng Tribute of Yu***	海岱惟青州。嵎夷既略，濰、淄其道。厥土白墳，海濱廣斥。厥田惟上下，厥賦中上。厥貢鹽絺，海物惟錯。岱畎絲、枲、鉛、松、怪石。萊夷作牧。厥篚檿絲。浮于汶，達于濟	The sea and (mount) Dai were the boundaries of Qing Zhou. (The territory of) Yu-yi was defined; and the Wei and Zi were made to keep their (old) channels. Its soil was whitish and rich. Along the shore of the sea were wide tracts of salt land. Its fields were the lowest of the first class, and its contribution of revenue the highest of the second. Its articles of tribute were salt, fine cloth of dolichos fibre, productions of the sea of various kinds; with silk, hemp, lead, pine trees, and strange stones, from the valleys of Dai. The wild people of Lai were taught tillage and pasturage, and brought in their baskets the silk from the mountain mulberry tree. They floated along the Wen, and so reached the Ji.	Fiber	Legge 1879
	荊河惟豫州。伊、洛、瀍、澗既入于河，滎波既豬。導菏澤，被孟豬。厥土惟壤，下土墳壚。厥田惟中上，厥賦錯上中。厥貢漆、枲，絺、紵，厥篚纖、纊，錫貢磬錯。浮于洛，達于河	The Jing (mountain) and the He were (the boundaries of) Yu Zhou. The Yi, the Luo, the Chan, and the Jian were conducted to the He. The (marsh of) Rong-bo was confined within its proper limits. The (waters of that of) Ge were led to (the marsh of) Meng-zhu. The soil of this province was mellow; in the lower parts it was (in some places) rich, and (in others) dark and thin. Its fields were the highest of the middle class; and its contribution of revenue was the average of the highest class, with a proportion of the very highest. Its articles of tribute were varnish, hemp, fine cloth of dolichos fibre, and the boehmerea. The baskets were full of chequered silks, and of fine floss silk. Stones for polishing sounding-stones were rendered when required. They floated along the Luo, and so reached the He.		

ESM1 Table S1, continued

禮記 Lǐ Jì<br>**the Classic of Rites**<br>**475 BC - 221 BC**	是月也，土潤溽暑，大雨時行，燒薙行水，利以殺草，如以熱湯。可以糞田疇，可以美土強。<br>[月令]	In this month the ground lies steaming and wet beneath the heats, for great rains are (also) continually coming. They burn the grass lying cut upon the ground and bring the water over it. This is as effectual to kill the roots as hot water would be; and the grass thus serves to manure the fields of grain and hemp, and to fatten the ground which has been but just marked out for cultivation.<br>[Proceedings of Government in the Different Months]	Fiber	Legge 1885<br>Li 1974
	斬衰，括髮以麻；為母，括髮以麻，免而以布。齊衰，惡笄以終喪。<br>[喪服小記]	When wearing the unhemmed sackcloth (for a father), (the son) tied up his hair with a hempen (band), and also when wearing it for a mother. When he exchanged this band for the cincture (in the case of mourning for his mother), this was made of linen cloth. (A wife), when wearing the (one year's mourning) of sackcloth with the edges even, had the girdle (of the same), and the inferior hair-pin (of hazel-wood), and wore these to the end of the mourning.<br>[Record of small matters in the dress of mourning]	Hemp clothbands mentioned 28 times through the book: used by mourners to wrap their head	
呂氏春秋<br>***Lǚshì Chūnqiū***<br>**Master Lü's Spring and Autumn Annals**<br>**By Lǚ Bùwéi**<br>**c. 247-239 BC**	是月也，天子始絺<br>[孟夏紀]	In this month, the Son of Heaven begins to dress in thin hemp clothes.<br>[Almanac for the First Month of Summer]	Fiber	Li 1974<br>Knoblock and Riegel 2000
	后妃率九嬪蠶於郊，桑於公田。是以春秋冬夏皆有麻枲絲繭之功，以力婦教也。<br>[上農]	The empress leads the nine consorts in nurturing the silkworm at the suburban altar and in picking the mulberry leaves in the common fields，and, as a result of this, there are the achievements of woven hemp and silk fabrics for all seasons.<br>[The Supreme Importance of Agriculture]		

ESM1 Table S1, continued

史記 Shì Jì<br>**Records of the Grand Historian**<br>**By Sima Qian**<br>**c. 109 -91 BC**	厥貢鹽絺，海物維錯，岱畎絲、枲、鉛、松、怪石，萊夷為牧，其篚酓絲。<br>[夏本紀]	Its tribute consisted of salt, fine cloth of dolichos fibre, and productions of the sea of various kinds, with silk, hemp, lead, pine-trees, and strange stones from the valleys of the Tai. The wild tribes of Lai were shepherds, and brought in their baskets silk from the mountain mulberry.<br>[Annals of the Xia]	Fiber; Grain?	Qian 1993
	泗水以北，宜五穀桑麻六畜<br>[列傳: 貨殖列傳]	The region north of the Zhe and Si rivers is suitable for growing the five types of grain, mulberries, and hemp, and for raising the six kinds of domestic animals.<br>[The Collective Biographies: The Money Makers]	Grain	
神農本草經 Shénnóng Běncǎo Jīng<br>**Divine Farmer's Classic of Materia Medica**<br>**c. 1st -2nd centuries AD**	麻贲味辛平。主五劳七伤，利五藏，下血，寒气，多食，令人见鬼狂走。久服，通神明，轻身。一名麻勃。麻子，味甘平，主补中益气，肥健不老神仙。生川谷。	*Ma Fen*[1] (*Herba Cannabis Sativae*) is acrid and balanced. It mainly treats the seven damages, disinhibits the five viscera, and precipitates the blood and cold *qi*. Taking much of it may make one behold ghosts and frenetically run about. Protracted taking may enable one to communicate with the spirit light and make the body light. The seed [*Semen Cannabis Sativae*] is sweet and balanced. It mainly supplements the center and boosts the *qi*. Protracted taking may make one fat, strong, and never senile.<br>*[Herba Cannabis Sativae*] is also called *Ma Bo* (Hemp Erection). It grows in rivers and valleys.	First clear written account for psychoactive use of cannabis	Li 1974: 446<br>Yang 1998<br>Liu 1999<br>Lu and Clarke 1995
尔雅注 Ĕr Yǎ zhù<br>**Commentary to Approach to correct expression**<br>**by Guō Pú c. 276-324 AD**	黂，枲实。枲麻。<br>苧，麻母。	枲 xi (*male* plant not producing seeds)<br><br>苴麻 jū má (interpreted by later scholars as the *female* plant producing seeds)	Dioecious nature of the plants, with recognition of male and female plants	Clarke and Merlin 2013: 141<br>Gao 1996

[1] Comment by translator Shou-zhong Yang (1998: 148): The current name of this medicinal is *Da Ma*. Its seed is currently called *Ma Ren* or *Da Ma Ren*. In modern clinical practice. Hemp Seeds are still in wide use. They are able to dredge wind qi, relax the spleen, moisten dryness, promote lactation, hasten delivery, and disinhibit urination and defecation.

ESM1 Table S1, continued

References

Biot, É. 1851. *Le Tscheou-li, ou rites des Tcheou*. Vol. 2. Une édition réalisée par Pierre Palpant, bénévole, Paris. Impr. nationale.

Clarke, R. and Merlin, M., 2013. *Cannabis: evolution and ethnobotany*. Berkeley: University of California Press.

Huang, H.T. 2000. *Science and Civilisation in China. Volume 6: Biology and Biological Technology. Part V: Fermentations and Food Science*. Cambridge: Cambridge University.

Gao R.G. 1996. Guo Pu. In Feng K.Z., Fu Q.S., (ed.), *Zhuzi baijia da cidian*. Shenyang: Liaoning renmin chubanshe.

Knoblock, J. and Riegel, J.K., 2000. *The Annals of Lü Buwei*. Stanford University Press.

Legge, J. 1876. *The Book of Poetry*. CreateSpace Independent Publishing Platform.

Legge, J. 1879. *The Sacred Books of China, part 1 of 6. PART I of the texts of Confucianism. The Shû King, the religious portions of the Shih King, the Hsiâo King*. Oxford: Clarendon Press.

Legge, J. 1885. *The Sacred Books of China, part 3 and 4 of 6. PART III, PART IV of the texts of Confucianism. The Lî Kî.* Oxford: Clarendon Press.

Li, Hui-Lin. 1974. An archaeological and historical account of cannabis in China. *Economic Botany* 28 (4):437-448.

Liu, Z. X. 1999. Further textual research into *mafen* as recorded in the Divine Farmer's Materia Medica. *J. Chin. Med. Mater.* 22, 211–212.

Lu, Xiaozhai, and Robert C Clarke. 1995. The cultivation and use of hemp (*Cannabis sativa* L.) in ancient China. *Journal of the International Hemp Association* 2 (1):26-30.

Qian, Sima & Burton, W. (translator) 1993. *Records of the grand historian: Han dynasty (Vol. 1 & 2).* Columbia University Press.

Yang, S. 1998. *The Divine Farmer's Materia Medica: a translation of the Shen Nong Ben Cao Jing*. Boulder, CO: Blue Poppy Enterprises, Inc.
